# Parenting and the decline of physical activity from age 9 to 15

**DOI:** 10.1186/1479-5868-8-33

**Published:** 2011-04-15

**Authors:** RH Bradley, S McRitchie, RM Houts, P Nader, M O'Brien

**Affiliations:** 1 School of Social and Family Dynamics, Arizona State University, 951 S. Cady Mall, Tempe, AZ, 85287, USA; 2 RTI International, Research Triangle Park, NC, 27709 USA; 3 Department of Psychology and Neuroscience, Duke University, 2020 West Main Street, Durham, NC, 27708 USA; 4 University of California - San Diego, 2855 Union Street, San Diego, CA, 92103 USA; 5 Department of Human Development and Family Studies, University of North Carolina at Greensboro, 248 Stone Building, Greensboro, NC, 27402 USA

## Abstract

**Background:**

There is a rapid decline in moderate-to-vigorous physical activity (MVPA) during middle childhood and adolescence. Information on the environmental factors implicated in this decline is limited. This study focuses on family factors associated with the rate of decline in objectively measured physical activity during middle childhood and adolescence.

**Methods:**

Longitudinal analysis of 801 participants from 10 US sites in the NICHD Study of Early Child Care and Youth Development whose data included accelerometer-determined levels of moderate-to-vigorous physical activity (MVPA) between ages 9 and 15 years, as well as family process, BMI and demographic information. The sample included an even split of boys (49%) and girls (51%), was predominantly white (77%), and contained about 26% low income and 19% single parent families. The outcome measure was mean MVPA. It was based on 4 to 7 days of monitored physical activity.

**Results:**

Boys with lower parental monitoring scores and more days of parental encouragement had significantly more minutes of MVPA at age 9 years. The effect of parental monitoring, however, was moderated by early puberty. High parental monitoring was associated with decreased activity levels for boys experiencing later puberty and increased activity for boy experiencing early puberty. Minutes of MVPA for boys living in the Midwest decreased at significantly faster rates than boys living in any other region; and boys in the South declined faster than boys in the West. Girls in the Midwest and South declined faster than girls in the West and Northeast. Among girls, more days of parental exercise and transportation to activities were associated with more MVPA per day at age 9. However, more parental transportation to activities and less monitoring was associated with faster linear declines in daughters' MVPA between the ages of 9 and 15 years. For girls who experienced puberty early, parental encouragement was associated with more MVPA.

**Conclusions:**

Parenting processes, such as monitoring and encouragement, as well as the parents' own level of physical activity, showed significant, but small, gender-specific associations with MVPA levels at age nine and the linear rate of decline in MVPA between ages 9 and 15.

## Background

Several recent large-scale studies demonstrate a dramatic decline in moderate to vigorous physical activity during middle childhood and adolescence [1-4]. This decline foreshadows low levels of physical activity in adulthood [5] and may contribute to a host of negative health outcomes [6]. Given the significance of this precipitous decline, several studies have attempted to identify factors that may reduce the rate of the decline. Studies of lifestyle (e.g., TV watching, sports participation), geographic (e.g., rates of neighborhood unemployment, availability of parks, ease of walking to school and recreational facilities), cultural (e.g., race, ethnicity) and psychological (e.g. stress tolerance, self-efficacy) factors have demonstrated only weak and inconsistent linkages to adequate participation in moderate-to-vigorous physical activity (MVPA) throughout childhood [7]. Some evidence suggests that family factors such as parental encouragement of physical activity and the parent's own level of activity may affect patterns of physical activity for school-age children; nonetheless, only very limited support exists for the idea that parental actions strongly influence the amount of MVPA [4,7].

This study aims to more intensively examine the connections between parenting and the decline of objectively measured moderate-to-vigorous physical activity from age 9 to age 15 years. It is a period of life when the impact of parents on children tends to wane [8-10]. Adolescent behavior (including engagement in physical activity) becomes more and more strongly associated with peer associations and behavior during middle childhood and adolescence [8]. It is also a period during which boys and girls undergo rapid maturation. Physical activity is a complex behavioral phenotype that is shaped by the interplay of biological and psychosocial factors, together with the physical environment [11]. Biological maturity appears to play a role in levels of physical activity [12]. However, findings to date have been inconsistent, partly as a consequence of how biological maturity was assessed and partly as a consequence of whether other key child characteristics (e.g., BMI) were controlled[13]. Because the rate of decline in MVPA is so rapid during this period, we will consider how parenting factors may be differentially relevant by age and gender, especially as there is some evidence for change in energy expenditure during the cross-over from prepubertal to post pubertal status [14]. Moreover, there is reason to believe that responses to parents may vary depending on pubertal timing. Specifically, there is gender intensification when signs of biological maturation appear, with both parent and child adjusting reactions to the other [15]. It is part of a larger shift in social relationships and attributions that occur with the transition.

Parental encouragement to be physically active is often cited as a way to promote physical activity among youth and adolescents, but evidence supporting this association is weak. Most studies have significant limitations in that they employ cross-sectional designs, use restrictive samples, or use parent reports of physical activity [16-22]. Evidence also suggests that children are more active if their parents are active; but results are mixed and details about this relation are lacking as a consequence of the samples examined and the measures of physical activity used [4,22-26]. For example, in their meta-analysis Pugliese and Tinsley [22] found that the average "effect" of parenting practices varied depending on whether physical activity was mechanically measured, obtained by self report or obtained using reports by others. Likewise, estimates varied as a consequence of what age was sampled and the sampling technique used [22]. Other family factors, such as parental provision of transportation and family time together, have been advanced as possible contributors to physical activity during middle childhood and adolescence, but the data in support of these are quite limited [4,18,22]. Finally, even though monitoring is connected to a variety of other child outcomes during adolescence [27], it remains rarely explored as a correlate of physical activity [28]. In view of evidence linking parental encouragement and monitoring to other lifestyle behaviors for adolescents [27] and evidence that positive parenting may be especially instrumental in maintaining adaptive behavior in early maturing children [29] we examine how pubertal status may moderate the effect of these behaviors on MVPA.

In overview, this study focuses on family factors associated with the decline in objectively measured physical activity during middle childhood and adolescence. We consider demographic factors (i.e., race/ethnicity, single parent status, family income) and biologic conditions (i.e., pubertal timing), but the primary focus is on five key parenting behaviors: (1) encouragement to be physically active, (2) provision of transportation to support physical activity, (3) parental monitoring of the child's behavior, (4) parent and child joint involvement in physical activity, and (5) the parent's own level of physical activity. Although there is conceptual and some empirical support for examining these factors, the literature does not currently support the assertion of strong hypotheses about relations between parenting practices and levels of MVPA.

## Methods

### Sample

The NICHD Study of Early Child Care and Youth Development is a multi-site study originally designed to determine the effects of non-maternal care on children's development. Participants were recruited in 1991 from designated community hospitals at 10 University-based data collection sites: (1) Little Rock, AR; (2) Irvine, CA; (3) Lawrence, KS; (4) Boston, MA; (5) Philadelphia and (6) Pittsburgh, PA; (7) Charlottesville, VA; (8) Seattle, WA; (9) Hickory and Morganton, NC; and (10) Madison, WI. Recruitment and selection procedures are described in detail [2] and study procedures are described on the NICHD website http://www.nichd.nih.gov/research/supported/seccyd/overview.cfm. Children were followed from birth to 15 years with a common study protocol, including interview, home, school, and neighborhood observations. For all study data collection protocols, including the accelerometer, human subjects institutional review boards at each university and the data coordinating center approved voluntary, written informed consents from participating families. All children gave verbal or implied assent by wearing the monitor. Healthy newborns, discharged within one week of birth, of English speaking mothers were recruited. When the target child was 2 weeks old, attempts were made to contact 3,015 families who met eligibility criteria to enlist their participation. Attempts to contact were unsuccessful for 512 families and 151 families were deemed ineligible because the child remained in the hospital more than seven days or the family planned to move. 641 families refused to participate and 1-month interviews could not be scheduled for 185 families for other reasons. Out of 1,526 families scheduled 1,364 families actually completed the 1-month home visit and became study participants. There were no significant differences between these 1,364 families and the 1990 U.S. population [30] based on ethnicity (80.3% white in the US population vs 80.4% in cohort) and household income (household income information available on 1,273 families; $36,520 in US population and $37,948 in cohort). The NICHD Study of Early Child Care and Youth Development cohort had a slightly higher percentage of married couple family households than the U.S. population (76.7% vs 74.2%, *p *= 0.04).

### Measures

#### Demographic and Child Characteristics

Child sex and race/ethnicity defined by the mother were recorded at 1 month. Race/ethnicity was coded as white or non-white (black, Hispanic, Asian, and other) and was originally collected to compare the characteristics of the study sample with the eligible population and because ethnicity is associated with patterns of child care use. Information on whether the mother had a partner/spouse living in the home was obtained by interview at ages 9, 11, 12, and 15. Family income was obtained by interview at age 9 and converted to an income-to-needs ratio based on federal poverty levels for each family size (ratio < 2.0 is considered low income) [30]. Data collection sites were grouped by region (Northeast: Pittsburgh and Philadelphia, Pennsylvania, and Boston, Massachusetts; South: Little Rock, Arkansas, Charlottesville, Virginia, and Hickory and Morgantown, North Carolina; Midwest: Lawrence, Kansas, and Madison, Wisconsin; and West: Irvine, California, and Seattle, Washington).

#### Early Puberty

Pubertal development was assessed using reports of daughter's age of menarche and annual physical exams of pubertal status using Tanner Criteria [31,32] at ages 9.5, 10.5, 11.5, 12.5, 13.5, 14.5 and 15.5 years. Early puberty was defined as pubertal onset prior to age 9.5 years for girls and prior to 10.5 years for boys [33].

#### Parental Monitoring

Parental monitoring was assessed via maternal interview at age 11 using nine questions from Stattin's Monitoring Measure [34]. Scores ranged from 1 to 4 with higher scores indicating increased parental knowledge of a child's whereabouts, activities, and associations. This measure has modest internal reliability (Cronbach's alpha = 0.63). Because this variable was extremely skewed, it was dichotomized into high (score = 4.0) versus low (score < 4) monitoring.

#### Parental Involvement in Physical Activity

Data on parental involvement in the child's physical activity was obtained at ages 9, 11, 12, and 15 years by asking the parent how many days per week he/she encouraged the child to be physically active, how many days per week he/she participated in a physical activity with the child, and how many days per week a family member provided the child transportation to an activity where the child was physically active.

#### Parental Exercise

Extent of parental exercise was obtained at ages 9, 11, 12, and 15 by asking the parent how many days per week he/she participated in at least 30 minutes, either continuous or in bouts of 10 minutes or more added together, of moderate intensity physical activity/exercise that was not work-related.

#### Monitored Physical Activity

The amount of physical activity each child engaged in across a typical week was measured using an accelerometer (Computer Science and Applications, Inc., Shalimar, FL) set so that it recorded minute-by-minute movement counts. Accelerometer determined physical activity was offered to the entire cohort at ages 9 (n = 1,098), 11 (n = 1,084), 12 (n = 1,064), and 15 (n = 1,009) years. Participation in wearing the monitor was high (80.1% at 9 years, 81.6% at 11 years, 70.7% at 12 years, and 68.9% at 15 years). The 2 reasons most often given for refusing to wear the monitors were inconvenience and concerns for the appearance around the waist of the 1.5 × 1.5 inch monitor. The slightly lower participation of adolescents wearing the monitor has been noted in other studies [35].

Participants wore the monitor on a belt around the waist during waking hours for 7 days, including two weekend days and five weekdays, excluding showering, bathing, water sports, or contact sports. These constraints on wearing the monitor (common to all accelerometer studies) resulted from manufacturers' suggestions and safety concerns (e.g., possible bruising or injury). Decisions about when to remove monitors were made by participants and coaches. Information from participant activity logs and patterns of observed counts indicate that the degree of underestimation of overall activity was minimal, and only for a few children during one or two days of the total activity recorded.

The number of counts recorded by the accelerometer was used to estimate the energy expended in moderate (3.0-5.9 metabolic equivalent tasks [METS]), vigorous (6.0-8.9 METS), and very vigorous (> 9.0 METS) activity, based on the age-specific equation of Freedson et.al [36]

Accelerometer data were downloaded to the same computer used to initialize them. A complete day of activity data was defined as extending from the first non-zero accelerometer count after 5 a.m. until one of the following criteria was met: (a) 60 consecutive minutes of zero counts after 9 p.m.; (b) 30 consecutive minutes of zero counts after 10 p.m.; or (c) the last non-zero count prior to midnight, whichever came first. Once the number of minutes for any given day was calculated, the total number of accelerometer counts was computed; then invalid days [too short a measurement time, implausible total count for the time recorded, zero counts, or any record shorter than 4 days] were flagged for removal. Rules for removal were based upon patterns observed from visual inspection of the data for 9 year old children.

After calculating the total number of minutes spent wearing the monitor and number of minutes spent in moderate, vigorous, or very vigorous activity, these minutes were summed to represent the total amount of time each child spent each day in moderate-to-vigorous physical activity (MVPA). The mean minutes per day of MVPA was calculated and used as the index of total activity for each day the monitor was worn.

Between-day intraclass reliability coefficients were calculated following the procedures outlined in Baumgartner [37]. Four-day reliabilities for minutes of MVPA averaged 0.75, 7-day reliabilities averaged 0.82.

### Statistical Procedures

All statistical analyses were conducted using SAS 9.1.3 (SAS Institute, Cary, NC) and all hypotheses were tested using 2-sided tests. Significant differences between children who had activity data and those who did not were determined for all categorical variables using χ^2 ^test.

To explore the effects of parenting on the child's average number of minutes of MVPA per day at each age as well as how parenting effects the change in minutes of MVPA between the ages of 9 and 15 years, we constructed quadratic growth curve models [38] separately for boys and girls using PROC MIXED (SAS Institute, Cary, NC). Such a model seemed useful given that MVPA reaches a kind of floor level as children move into mid-adolescence. A quadratic model has been previously shown to be a good fit for modeling the change in activity between the ages of 9 and 15 years [2]. Modeling the change in minutes of MVPA using growth curves allowed us to calculate the mean trajectory of minutes of MVPA as well as an estimation of each child's individual trajectory. All models used restricted maximum likelihood estimates, which result in less biased estimates since both fixed and random effects are treated as unknowns. We tested each model with a homoscedastic error structure against the model with a heteroscedastic error structure using the likelihood ratio test and determined that the heteroscedastic error structure improved the model fit for both boys and girls (*p *<.001). As a result, models assumed unequal residual variance and an unstructured covariance matrix.

Age was treated as a continuous variable and centered at age 9. Demographic and biological characteristics (i.e., race/ethnicity, low income, single parent family, region, early puberty) were used as control variables. Parental monitoring at age 11 was treated as a categorical time invariant covariate with low parental monitoring serving as the reference group. The number of days per week the parent encouraged the child to be physically active, number of days per week the parent participated in a physical activity with the child, number of days a family member provided the child with transportation to an activity where he/she was physically active, and the reported number of days the parent spent in MVPA were treated as continuous time-varying covariates that were grand mean centered. All interaction terms were created using the centered variables [39]. Children who had valid accelerometer data were excluded from the model if they were missing data on any of the covariates included in the model.

The model for girls included a random intercept and linear slope. The model for boys only included a random intercept because the intercept and slope were highly correlated and the variance of the centered age parameter was not significant, leading to convergence problems. The high negative correlation observed between the intercept and linear slope in the boy's model indicated that the linear rate of decline in average minutes of MVPA was greater for those boys who had more minutes of MVPA at age 9.

## Results

A total of 396 boys and 405 girls had valid activity measurements for at least 1 of the 4 assessments (9, 11, 12, and 15 years) and were included in these analyses. As shown in Table 1, 76% of the boys in the sample were white, 25% low income, 18% experienced early puberty (pubertal onset prior to age 10.5 years), and 43% experienced high parental monitoring at age 11. 79% of the girls in the sample were white, 27% low income, 22% experienced early puberty (pubertal onset prior to 9.5 years), and 50% experienced high parental monitoring at age 11.

**Table 1:** Descriptive Characteristics of the Time Invariant Covariates for the NICHD Study of Early Child Care and Youth Development

	Boys (N = 396)		Girls (N = 405)	
		
	Count	%	Count	%
Race/Ethnicity^a^				
Nonwhite	95	23.99	87	21.48
White	301	76.01	318	78.52
Low Income at Age 9				
Yes	99	25.00	110	27.16
No	297	75.00	295	77.53
Puberty				
Early	71	17.93	91	22.47
Later	325	82.07	314	77.53
High Monitoring - Age 11^b^				
Yes	169	42.68	203	50.12
No	227	57.32	202	49.88
Region of Country				
Midwest	73	18.43	100	24.69
Northeast	108	27.27	116	28.64
South	116	29.29	102	25.19
West	99	25.00	87	21.48

Abreviations: NICHD = National Institute of Child Health and Human Development

^a ^Race/Ethnicity was defined by the mother and recorded at the 1-month interview. Nonwhite includes African American, Hispanic, Asian and other

^b ^Possible range of Parental Monitoring Score is 1 to 4. High parental monitoring was defined as a score of 4.

The initial sample for the NICHD Study of Early Child Care and Youth Development included 1364 children see NICHD Earl Child Care Research Network [40] for a more complete description of the sampling design and sample characteristics. The 396 boys who had at least one valid accelerometer reading and were included in this analysis sample were not significantly different from the 309 other boys from the original sample who were not included in the analysis sample for this study in terms of race/ethnicity, percentage living in low income families at 1 month, and early puberty. Likewise, the 405 girls who were included in the analysis sample were not significantly different from the other 254 girls who were in the original sample in terms of race/ethnicity and early puberty. However, there was a significant difference (*p *= 0.007) for the percentage of girls living in a low income family. Fifty-two percent of girls not included in the analysis sample were living in low-income families compared to 41% of those girls who were included in the analysis sample.

### Results of Growth Curve Analyses

Table 2 shows the findings for the growth curve models relating parenting to activity for boys and girls. At age 9, boys and girls spent approximately 3.0 and 2.7 hours per day, respectively, in MVPA. Both boys and girls showed significant linear declines in the average minutes of MVPA per day between age 9 and age 15; however, the significant quadratic effect (i.e., Age × Age) indicates that the linear rate of decline leveled off as children entered adolescence.

**Table 2:** Growth Curve Models Examining How Parenting Relates to Changes in Average Minutes Per Day of MVPA Between Ages 9 and 15 Years

	Boys (N = 396)			Girls (N = 405)		
		
	*p*-value	Estimate	ES^a^	*p*-value	Estimate	ES^a^
Intercept @ Age 9		183.51			164.04	
White (0) vs non-White (1)	0.77	1.47	0.01	0.46	3.94	0.04
Low-Income @ Age 9: Yes (1), No (0)	0.049*	10.15	0.07	0.17	7.07	0.07
Single-Parent Family: Yes (1), No (0)	0.19	6.58	0.04	0.86	0.83	0.01
Region	0.12			0.11		
Midwest		14.07	0.08		13.61	0.12
Northeast		5.21	0.03		4.85	0.04
South		2.95	0.02		9.74	0.08
West (reference)						
Puberty: Early (1), Later (0)	0.19	-6.62	0.06	0.89	0.55	0.01
BMI Percentile	<0.001*	-0.41	0.19	0.023*	-0.16	0.10
High Level Parental Monitoring: Yes (1), No (0)	0.046*	-8.70	0.07	0.74	-1.48	0.02
Parental Encouragement: Days per Week	0.002*	2.73	0.11	0.62	0.38	0.02
Parental Participation: Days per Week	0.80	-0.30	0.01	0.18	1.43	0.05
Parental Transportation: Days per Week	0.042*	2.55	0.07	<0.001*	4.99	0.14
Parental Exercise: Days per Week	0.44	0.75	0.03	.01*	2.22	0.09
Low-Income × Parental Transportation	0.71	-0.81	0.01	<0.001*	-7.82	0.14
Early Puberty × Parental Encouragement	0.01*	-5.22	0.09	0.29	-1.73	0.05
Early Puberty × High Parental Monitoring	0.14	14.70	0.06	0.30	-8.07	0.05
Linear Slope (Age)	<0.001*	-35.57	0.65	<0.001*	-37.73	0.76
Age × White vs Non-White	0.89	0.15	0.01	0.81	0.26	0.01
Age × Low-Income	0.60	-0.60	0.03	0.33	-1.04	0.05
Age × Single Parent Family	0.86	0.21	0.01	0.64	-0.50	0.02
Age × Region	<0.001*			<0.001*		
Age × Midwest		-5.42	0.19		-3.61	0.17
Age × Northeast		-0.71	0.03		-0.21	0.01
Age × South		-2.40	0.10		-3.67	0.17
Age × West (reference)						
Age × BMI Percentile	0.003*	0.05	0.14	0.06	0.03	0.10
Age × High Parental Monitoring	0.27	-1.03	0.05	0.69	0.37	0.02
Age × Parental Encouragement	0.08	-0.35	0.07	0.49	-0.14	0.03
Age × Parental Participation	0.22	0.37	0.05	0.37	-0.24	0.04
Age × Parental Transportation	0.21	-0.34	0.05	0.003*	-0.76	0.12
Age × Parental Exercise	0.38	-0.21	0.04	0.40	-0.19	0.04
Age × Low-Income × Parental Transportation	0.64	-0.23	0.02	0.013*	1.18	0.11
Age × Early Puberty × Parental Encouragement	0.034*	1.02	0.09	0.014*	1.04	0.12
Age × Early Puberty × Parental Monitoring	0.44	1.49	0.04	0.32	1.42	0.06
Quadratic Slope (Age × Age)	<0.001*	2.68	0.36	<0.001*	2.84	0.51

Child's age is centered at age 9 years; BMI percentile, parental encouragement, parental participation, parental transportation, and parental exercise are grand mean centered.

^a ^Effect size for 2-level categorical variables was calculated using Cohen's d and effect size for region was calculated using Cohen's f. Cohen, J (1988). Statistical power analysis for the behavioral sciences (2nd ed.). New York, NY: Lawrence Erlbaum Associates.

* Statistically significant

#### Boys' Activity

**Figure 1:**
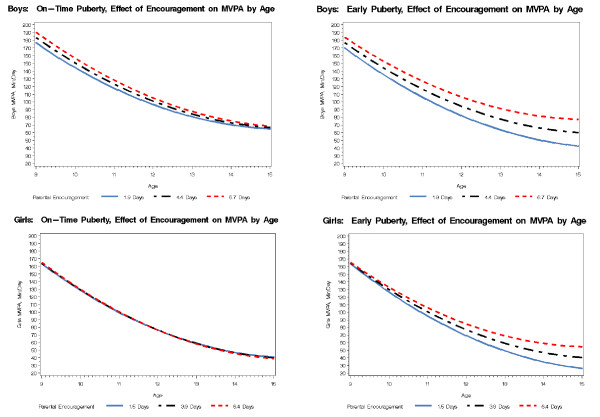
**The effect of parental encouragement on minutes of MVPA by pubertal status**. Included as separate.doc file.

Boys' minutes of MVPA at age 9 (i.e., the growth curve intercept) were associated with family income, BMI percentile, and three parenting variables (i.e., high parental monitoring, transportation to activities, and parental encouragement). Early puberty moderated the effect of parental encouragement. Specifically, boys whose families had low-income exhibited 10 more minutes of MVPA per day at age 9 than did boys whose families had higher incomes. Furthermore, there was about 4 minutes less MVPA at age 9 for each 10 percentile increase in BMI above the mean 66^th ^percentile (See Figure 1).

When boys were age 9, each additional day (above the mean level of 2.6 days) of parental transportation to activities was associated with about 3 extra minutes of MVPA. By contrast, higher levels of parental monitoring were associated with lower levels of MVPA (about 9 minutes per day on average). Beyond the mean of 4.4 days of encouragement, each additional day of parental encouragement to be active was associated with about 3 more minutes of MVPA for boys who experienced on-time puberty; however, it was associated with about 2 minutes per day less MVPA for boys who experienced early puberty.

Minutes of MVPA per day for boys decreased significantly between ages 9 and 15. At age 9, the linear rate of decline was 35.6 minutes of MVPA per day per year. The linear rate of decline leveled off as boys aged (e.g., at age 15, it was 19.5 minutes of MVPA per day per year). Region of the country, BMI percentile and the interaction between early puberty and parental encouragement to be active were significantly associated with linear declines in boys' MVPA between ages 9 and 15. Specifically, region of the country was associated with differences of about 6 minutes per day per year of MVPA. Boys living in the Midwest declined faster than boys living in any other region; and boys living in the South declined faster than boys living in the West. Above the 66^th ^percentile (the mean for the sample), every 10 percentile increase in BMI was associated with less steep declines of about 1 minute of MVPA per day. Each additional day of parental encouragement to be active above the mean of 4.4 days was associated with 1 minute per day slower decline in MVPA, but only for boys who experienced early puberty.

#### Girls' Activity

At age 9, girl's minutes of MVPA was associated with BMI and two parenting variables (transportation to activities and parental exercise). Family income moderated the relation between activity and transportation to activities. Specifically, each 10 percentile increase in BMI was associated with 2 fewer minutes of MVPA per day at age 9.

Beyond the mean of 2.5 days of parental exercise, each additional day that parents exercised was associated with girls spending approximately 2 more minutes per day doing MVPA. For low income girls, each additional day of transportation to activities above the mean 2.3 days was associated with 3 fewer minutes of MVPA per day at age 9. In contrast, for higher income girls, each additional day of transportation to activities was associated with approximately 5 additional minutes of MVPA per day.

For girls, minutes of MVPA per day also decreased significantly between ages 9 and 15. At age 9, the linear rate of decline was 37.7 minutes of MVPA per day per year. This linear rate of decline leveled off as girls aged (e.g., at age 15, it was 20.7 minutes per day per year). Region of the country, transportation to activities, the interaction between transportation and low-income, and the interaction between early puberty and encouragement were all associated with the linear changes in girls' minutes of MVPA between ages 9 and 15 (See Figure 1). Mean MVPA across regions varied by 4 minutes per day per year. Girls living in the Midwest and South declined faster in their MVPA than did girls living in the West and Northeast. The interaction between low income and transportation to activities that was noted at age 9 became non-significant over time. By the time girls were age 15, transportation exerted little or no influence (regardless of income level) on MVPA. Encouragement to be active was unrelated to MVPA for girls who experienced on-time puberty. Nonetheless, girls who experienced early puberty were more active as adolescents when they received more encouragement from their parents to be active.

## Discussion

The role parents play in the trajectory of their children's objectively measured physical activity from age 9 to age 15 was examined for more than 800 children. As expected, relations differed for boys and girls and differed somewhat as a function of age and pubertal timing. Although parental behavior was associated with the level of MVPA for both boys and girls, in neither case did parenting factors show a strong association with the rate of decline in MVPA. The small effect sizes observed for parenting factors is typical in studies of physical activity from middle childhood onward [20,26,41,42]. As previously shown, the minutes of moderate-to-vigorous physical activity per day declined dramatically from age 9 to age 15 years for both boys and girls [2]. The extent to which parents themselves engaged in physical activity mattered for 9 year old girls, but not boys. Prior studies have shown that parental participation was sometimes a factor in children's levels of participation [4,19-23,43,44]; but our study indicates that it does not slow the rate of decline in MVPA for girls (indeed, girls whose parents exercised more actually show steeper rates of decline such that there was less difference at age 15 than at age 9). Moreover, the findings showed that parental exercise plays little, if any, role in the rate of decline for boys. Other studies done exclusively with girls have also shown that the level of parental involvement in physical activity is associated with physical activity in younger girls [45]. The differences in findings across studies likely reflect the more limited samples used in most studies and the failure to examine objectively measured physical activity in some [46].

By contrast, parental encouragement to be active seemed to matter for 9 year old boys, but not much for girls. Again, however, the rate of decline for boys whose parents encouraged them to exercise was steeper so that there was less difference by age 15. Some prior studies have shown that encouragement plays a role in adolescent MVPA [15-18,20,22]; but, in our study, it did not play a significant a role in girls' level of activity. Moreover, for our boys the role that encouragement plays in MVPA appears to vary depending on whether they enter puberty early or late. For those who entered puberty early, encouragement was associated with a lower rate of decline in MVPA. Contrary to what happens in response to parental encouragement, the more parents monitored boys, the more rapid the decline of MVPA. Perhaps this effort to control boys' behavior, though intended as protection [17], had the unintended consequence of also reducing involvement in physical activity [32].

For girls, the role of parental involvement also appears to be complex. For example, providing transportation for girls to places that afford opportunities for physical activity was associated with more MVPA when girls were younger; but the relation waned as girls aged. That said, there was a distinct difference in low income and high income families. In high income families, more transportation was associated with increasing MVPA - perhaps indicative of parental efforts to support girls' athletic interests. The apparent declining significance of parental transportation generally may reflect the increasing importance of peer associations in adolescents' lives and the fact that girls report having relatively few friends who are actively engaged in physical activities and sports [8].

Pubertal timing was not a significant factor in level of MVPA for either boys or girls. Such a finding is not surprising in that findings pertaining to most markers of biological maturity have been mixed [13]. Other studies that controlled for age and BMI like we did have frequently found non-significant associations between biological maturity and physical activity [47].

This study is limited in a number of respects. First, although the sample is large and diverse, it is not nationally representative. Second, the data set does not contain a measure of the extent to which parents monitored physical activity except at age 11 - albeit, monitoring of most particular activities tends to be relatively stable. Third, we relied on relatively brief parent report measures for several key constructs, parental level of physical activity being a good example. Although the measures used in this study are similar to those used in other studies, more precise estimates of relations may have emerged if we had more extensive coverage of the constructs. Fourth, the data set did not contain information on the availability of nearby parks, playgrounds and other open spaces. Finally, the complex relation observed between parental encouragement to be active and actual levels of physical activity bears greater scrutiny and further examination [47], particularly in view of the fact that we did not have data on either prior to age 9. Thus, it is impossible to determine the actual direction of causal influence. The same limitation applies to other associations between parental behavior and MVPA in this study. These limitations acknowledged, our study is rare in having repeated measures of monitored physical activity and parenting processes for such a large and diverse sample, making it possible to more intensively examine relations between parenting practices and the level of physical activity children engage in during a period of life when moderate to vigorous activity dramatically declines.

## Conclusions and Implications

The decline in physical activity during middle childhood and adolescence is pervasive. No one factor appears to account for very much of this decline. Indeed, lower levels of MVPA appears characteristic of the lifestyles of youth and adolescents in most developed countries with no obvious set of demographic, geographic, personality or family factors accounting for more than a small fraction of the decline from early childhood to adolescence and beyond. This pattern is generally consistent with the expectancy-value theory of adolescent motivation [48]. Specifically, compared to spending time in other activities, spending time exercising is not highly valued by most adolescents. It is not tightly connected to their personal and social identities. For adolescent girls in particular, there is also evidence that the disinclination to be physically active connects to a sense that they are not competent to engage in many athletic activities [49]. Relatedly, there tend to be few social incentives for being physically active (i.e., the peer affiliations of most adolescents do not include many highly active members). That said, findings from this study suggest that parental encouragement to be active and the parent's own level of activity probably do play a small part. There is also evidence that general social support from families may foster higher levels of physical activity during adolescence^44^. Thus, a focus on helping parents be more deliberate and consistent - perhaps even innovative -- in such actions might be avenues for interventions that could have some impact on the decline. In some respects the complex pattern of relations observed between adolescent MVPA and parental transportation was unexpected. Given that parents would generally be encouraged to provide transportation to places that afford opportunities for physical activity, further examination of this relation is warranted. Likewise, it might be useful to investigate ways that parents might attempt to connect physical activity to other things that adolescents consider valuable. The theory of planned behavior, with its emphasis on volitional intention, suggests that such avenues would at least be worth exploring [50]. Finally, a deeper understanding of how parents can affect children's engagement in physical activity may come when studies can combined detailed measures of the family environment with detailed measures of the built environment [51], as access to different facilities and spaces offer different sets of opportunities for parental action and child investment in physical activity.

## Competing interests

The authors declare that they have no competing interests.

## Authors' contributions

RHB made a substantial contribution to the overall conception of the study, was PI at one of the 10 data collection sites, helped guide the analyses and interpretation of findings and helped draft the manuscript.

SMR was the primary statistician, helped interpret the findings and helped draft the Methods and Results sections of the manuscript. She also helped in revising the manuscript.

RMH also helped with statistical analysis, helped in interpreting findings and in revising the manuscript. She also established the principle way of aggregating MVPA data for purposes of analysis.

PN helped design the portion of the study dealing with physical activity, including training data collectors on the procedures for gathering data. He assisted in the overall conception of the study and in interpretation of findings.

MO'Bhelped in crafting the overall conception of the study, was PI at one of the 10 data collection sites, and helped in interpreting the findings.

## References

[B1] Troiano RP Berrigan D Dodd KW Masse LC Tilert T McDowell M Physical activity in the United States measured by accelerometer Med Sci Sports Exerc 2008 40 181 188 18091006 10.1249/mss.0b013e31815a51b3

[B2] Nader PR Bradley RH Houts RM McRitchie SL O'Brien M Moderate-to-vigorous physical activity from ages 9 to 15 years JAMA 2008 300 295 305 10.1001/jama.300.3.295 18632544

[B3] Riddoch CJ Bo Andersen L Wedderkopp N Harro M Klasson-Heggebø L Sardinha LB Cooper AR Ekelund U Physical activity levels and patterns of 9- and 15-yr-old European children Med Sci Sports Exerc 2004 36 86 92 10.1249/01.MSS.0000106174.43932.92 14707773

[B4] Terzian J Moore KA Physical inactivity in U.S. adolescents: family, neighborhood, and individual factors Child Trends Res Brief http://www.childtrends.org

[B5] Barnekow-Bergkvist M Hedberg G Janlert U Jansson E Prediction of physical fitness and physical activity level in adulthood by physical performance and physical activity in adolescence - an 18-year follow-up study Scand J Med Sci Sports 1998 8 299 308 10.1111/j.1600-0838.1998.tb00486.x 9809389

[B6] Anderson LB Harro M Sardinha LB Froberg K Kekland U Brage S Anderssen SA Physical activity and clustered cardiovascular risk in children: a cross-sectional study (The European Youth Heart Study) Lancet 2006 368 299 304 10.1016/S0140-6736(06)69075-2 16860699

[B7] Sallis JF Simons-Morton BG Stone EJ Corbin CB Epstein LH Faucette N Iannotti RJ Killen JD Klesges RC Petray CK Rowland TW Taylor WC Determinants of physical activity and interventions in youth Med Sci Sports Excerc 2000 24 S248 S257 1625550

[B8] King KA Tergerson JL Wilson BR Effect of social support and adolescents' perceptions of and engagement in physical activity J Phys Activity Health 2008 5 374 384 10.1123/jpah.5.3.374 18579916

[B9] Pettit GS Keiley MK Laird RD Bates JE Dodge KA Predicting the developmental course of mother-reported monitoring across childhood and adolescence from early proactive parenting, child temperament, and parents' worries J Fam Psych 2007 21 206 217 10.1037/0893-3200.21.2.206 PMC2791369 17605543

[B10] Salvy SJ Bowker JW Roemmich JN Romero N Kieffer E Paluch R Epstein LH Peer influence on children's physical activity: an experience sampling study J Pediatr Psych 2008 33 39 49 10.1093/jpepsy/jsm039 PMC2706580 17525088

[B11] Eisenmann JC Wickel EE The biological basis of physical activity in children: revisited Pediatr Exerc Sci 2009 21 257 272 19827450 10.1123/pes.21.3.257

[B12] Baker BL Birch LL Trost SG Davison KK Advanced pubertal status at age 11 and lower physical activity in adolescent girls J Pediatr 2007 488 493 17961691 10.1016/j.jpeds.2007.04.017 PMC2531153

[B13] Sherar LB Cumming SP Eisenmann JC Baxter-Jones AD Malina RM Adolescent biological maturity and physical activity: biology meets behavior Pediatr Exerc Sci 2010 22 332 349 20814031 10.1123/pes.22.3.332

[B14] Goran MI Gower BA Nagy TR Johnson RK Developmental changes in energy expenditure and physical activity in children: evidence for a decline in physical activity in girls before puberty Pediatrics 1998 101 887 891 10.1542/peds.101.5.887 9565420

[B15] Crouter AC Manke BA McHale SM The family context of gender intensification in early adolescence Child Dev 1995 66 317 329 10.2307/1131580 7750368

[B16] Kleges RC Malott JM Boschee PF Weber JM The effects of parental influences on children's food intake, physical activity, and relative weight Int J Eat Disorders 1986 5 35 346 10.1002/1098-108X(198601)5:1<35::AID-EAT2260050104>3.0.CO;2-F

[B17] McKenzie TL Sallis JF Nader PR Patterson TL Elder JP Berry CC Rupp JW Atkins CJ Buono MJ Nelson JA BEACHES: an observational system for assessing children's eating and physical activity and associated events J Appl Behav Anal 1991 24 141 151 10.1901/jaba.1991.24-141 2055797 PMC1279555

[B18] Beets JQ Vogel R Forlaw L Pitetti KH Cardinal BJ Social support and youth physical activity: the role of provider and type Amer J Health Behav 2006 30 278 289 10.5555/ajhb.2006.30.3.278 16712442

[B19] Bauer KW Nelson MC Boutelle KN Neumark-Sztainer D Parental influences on adolescents' physical activity and sedentary behavior: Longitudinal findings from Project EAT-II Int J Beh Nutr Phys Act 2008 5 12 10.1186/1479-5868-5-12 PMC2265744 18302765

[B20] Trost SG Sallis JF Pate RR Freedson PS Taylor WC Dowda M Evaluating a model of parental influence on youth physical activity Am J Prev Med 2003 25 277 282 10.1016/S0749-3797(03)00217-4 14580627

[B21] Krosnick JA Judd CM Transitions in social influence at adolescence: Who induces cigarette smoking? Dev Psych 18 359 364 10.1037/0012-1649.18.3.359

[B22] Pugliese J Tinsley B Parental socialization of child and adolescent physical activity: a meta-analysis J Fam Psych 2007 21 331 343 10.1037/0893-3200.21.3.331 17874918

[B23] Moore LL Lombardi MJ White JL Campbell SA Oliveria SA Ellison RC Influence of parents' physical activity levels on the activity levels of young children J Pediatr 1991 118 215 219 10.1016/S0022-3476(05)80485-8 1993947

[B24] Ross JG Pate RR The National Children and Youth Fitness Study: a summary of findings J Phys Educ Recreat Dance 1987 58 51 56

[B25] Sallis JF Patterson TL McKenzie TL Nader PR Family variables and physical activity in preschool children J Dev Behav Pediatr 1988 9 57 61 10.1097/00004703-198804000-00001 3366911

[B26] Kimiecik JC Horn TS Parental beliefs and children's moderate-to-vigorous physical activity Res Q Exercise Sport 1998 69 163 175 10.1080/02701367.1998.10607681 9635330

[B27] Dick AM Viken R Purcell S Pulkkinnen L Rose RJ Parental monitoring moderates the importance of environmental influences on adolescent smoking J Abnorm Psych 2007 116 213 218 10.1037/0021-843X.116.1.213 PMC1807367 17324032

[B28] Ornelas IJ Perreira KM Ayala GX Parental influences on adolescent physical activity: a longitudinal study Int J Behav Nutri Phys Act 2007 4 3 10.1186/1479-5868-4-3 PMC1805507 17274822

[B29] Mrug S Elliott M Gilliland MJ Grunbaum JA Tortolero SR Cuccaro P Schuster M Positive parenting and early puberty in girls Arch Pediatr Adolesc Med 2008 162 781 786 10.1001/archpedi.162.8.781 18678812

[B30] US Census Bureau 1990 Census http://www.census.gov/main/www/cen1990.html Accessed May 31, 2008

[B31] Marshall WA Tanner JM Variations in the pattern of pubertal changes in girls Arch Disease Child 1969 44 291 303 10.1136/adc.44.235.291 PMC2020314 5785179

[B32] Marshall WA Tanner JM Variations in the pattern of pubertal changes in boys Arch Disease Child 1970 45 13 21 10.1136/adc.45.239.13 PMC2020414 5440182

[B33] Belsky J Steinberg LD Houts RM Friedman SL DeHart G Cauffman E Roisman GI Halpern-Felsher BL Susman E NICHD Early Child Care Research Network Family rearing antecedents of pubertal timing Child Dev 2007 78 1302 1321 10.1111/j.1467-8624.2007.01067.x 17650140

[B34] Stattin H Kerr M Parental monitoring: a reinterpretation Child Dev 2000 71 1072 1085 10.1111/1467-8624.00210 11016567

[B35] Frank GC Nader PR Zive MM Broyles SL Brennan JJ Retaining children and families in community research; lessons from the Study of Children's Activity and Nutrition (SCAN) J Sch Health 2003 73 2 51 57 10.1111/j.1746-1561.2003.tb03571.x 12643019

[B36] Freedson P Prober D Jasnz KJ Calibration of accelerometer output for children Med Sci Sports Exerc 2005 37 11 suppl S523 S530 10.1249/01.mss.0000185658.28284.ba 16294115

[B37] Baumgartner TA Safrit MJ, Wood TM Norm-referenced measurement: reliability Measurement Concepts in Physical Education and Exercise Science 1989 Champaign, IL:Human Kinetcis Books 45 72

[B38] Diggle PJ Heagerty P Liang KY Zeger SL Analysis of Longitudinal Data 2002 2 Oxford, England: Oxford University Press

[B39] Aiken LS West SG Multiple Regression: Testing and Interpreting Interactions 1991 Thousand Oaks, CA:Sage

[B40] National Institute of Child Health and Human Development Early Child Care Research Network Nonmaternal care and family factors in early development: an overview of the NICHD study of early child care Appl Dev Psych 2001 22 457 492 10.1016/S0193-3973(01)00092-2

[B41] Sallis JF Prochaska JJ Taylor WC A review of correlates of physical activity of children and adolescents Med Sci Sports Exerc 2000 32 963 975 10.1097/00005768-200005000-00014 10795788

[B42] Ferreira I van der Horst K Wendel-Vos W Kremers S van Lenthe FJ Brug J Environmental correlates of physical activity in youth - a review and update Obes Rev 2006 8 129 154 10.1111/j.1467-789X.2006.00264.x 17300279

[B43] Hood MY Moore LL Sundarajan-Ramamuriti A Singer M Cupples LA Ellison RC Parental eating attitudes and the development of obesity in children. The Framingham Children's Study Int J Obes 2000 24 1319 1326 10.1038/sj.ijo.0801396 11093294

[B44] Crawford D Cleland V Timperio A Salmon J Andrianopoulos N Roberts R Baur L Ball K The longitudinal influence of home and neighborhood environments on children's body mass index and physical activity over 5 years: the CLAN study Int J Obes 2010 10.1038/ijo.2010.57 20351728

[B45] Davison KK Cutting TM Birch LL Parents' activity-related parenting practices predict girls' physical activity Med Sci Sports Excerc 2003 35 1589 1595 10.1249/01.MSS.0000084524.19408.0C PMC2530913 12972881

[B46] Gustafson SL Rhodes REE Parental correlates of physical activity in children and adolescents Sports Med 2006 36 79 97 10.2165/00007256-200636010-00006 16445312

[B47] Drenowatz C Eisenmann JC Pfeiffer KA Wickel EE Gentile D Walsh D Maturity-related difference in physical activity among 10- to 12-year-old girls Am J Hum Biol 2010 22 18 22 10.1002/ajhb.20905 19309682

[B48] Fredericks JA Eccles JS Weiss MR Parental influences on youth involvement in sports Developmental Sport and Exercise Psychology: a Lifespan Perspective 2004 Morgantown, WA: Fitness Information Technology, Inc 145 164

[B49] Davison KK Schmatz DL Downs D Hop, skip ...no! Explaining adolescent girls' disinclination for physical activity Ann Beh Med 2010 39 290 302 10.1007/s12160-010-9180-x PMC5542820 20393818

[B50] Chatzisarantis JLD Frederick C Biddle SJH Hagger JS Smith S Influences of volitional and forced intentions on physical activity and effort within the theory of planned behaviour J Sports Sci 2007 25 699 709 10.1080/02640410600818523 17454537

[B51] Boone-Heinonen J Evenson KR Song Y Gordon-Larsen P Built and socioeconomic environments: patterning and associations with physical activity in U.S. adolescents Int J Beh Nutr Phys Act 2010 7 45 10.1186/1479-5868-7-45 PMC3152773 20487564

